# Soil C and N statuses determine the effect of maize inoculation by plant growth-promoting rhizobacteria on nitrifying and denitrifying communities

**DOI:** 10.1038/s41598-017-08589-4

**Published:** 2017-08-21

**Authors:** Alessandro Florio, Thomas Pommier, Jonathan Gervaix, Annette Bérard, Xavier Le Roux

**Affiliations:** 1 Laboratoire d’Ecologie Microbienne LEM, INRA UMR 1418, CNRS UMR 5557, Université Lyon 1, Université de Lyon, F-69622 Villeurbanne Cedex, France; 2 INRA, UMR1114 EMMAH, Site Agroparc, 84914 Avignon, France

## Abstract

Maize inoculation by *Azospirillum* stimulates root growth, along with soil nitrogen (N) uptake and root carbon (C) exudation, thus increasing N use efficiency. However, inoculation effects on soil N-cycling microbial communities have been overlooked. We hypothesized that inoculation would (i) increase roots-nitrifiers competition for ammonium, and thus decrease nitrifier abundance; and (ii) increase roots-denitrifiers competition for nitrate and C supply to denitrifiers by root exudation, and thus limit or benefit denitrifiers depending on the resource (N or C) mostly limiting these microorganisms. We quantified (de)nitrifiers abundance and activity in the rhizosphere of inoculated and non-inoculated maize on 4 sites over 2 years, and ancillary soil variables. Inoculation effects on nitrification and nitrifiers (AOA, AOB) were not consistent between the three sampling dates. Inoculation influenced denitrifiers abundance (*nirK*, *nirS)* differently among sites. In sites with high C limitation for denitrifiers (i.e. limitation of denitrification by C > 66%), inoculation increased *nirS*-denitrifier abundance (up to 56%) and gross N_2_O production (up to 84%), likely due to increased root C exudation. Conversely, in sites with low C limitation (<47%), inoculation decreased *nirS*-denitrifier abundance (down to −23%) and gross N_2_O production (down to −18%) likely due to an increased roots-denitrifiers competition for nitrate.

## Introduction

The rhizosphere provides a peculiar environment where a huge variety of positive, negative and neutral interactions between roots and microorganisms occur^1^. Such interactions can significantly influence plant growth as well as the functioning, the abundance and the diversity of rhizospheric microbial communities^2^. Beneficial interactions are known to be established by plant growth-promoting rhizobacteria, PGPRs, with host plants through several mechanisms, including associative N_2_ fixation, phosphate solubilization or phytosiderophore production^3, 4^. This can result in improved root growth^5, 6^, increased number and length of lateral roots^7^, as well as an increased root and shoot biomass^8, 9^ and physiology^10^. The better root development induced by inoculation can consequently enhance nutrient^11^ and water^12^ uptake by plant, stimulate ion transport systems in root^13^ and increase the amount of root carbon, C, exudation^14, 15^.

*Azospirillum spp*. are well-known PGPRs that are able to colonize the roots of many crop plant species including maize^16, 17^. These PGPRs produce phytohormones that can promote root growth and improve nutrient and water absorption by plants^18–21^. In particular, inoculation of cereal crops by PGPRs like the well-studied *Azospirillum lipoferum* CRT1 is often expected to improve crop capacity to retrieve mineral nitrogen, N, from soil. This could pave the way for improving the sustainability of these cropping systems under low N inputs conditions^8^. However, inoculated plants could differently affect N dynamics in their rhizosphere, thus influencing the levels and types of mineral N forms available and possibly N losses from soil through leaching of nitrate, NO_3_^−^, or emission of nitrous oxide, N_2_O, a potent greenhouse gas^22^.

Understanding the success or failure of crop plant inoculation by PGPRs regarding N economy thus requires understanding the complex interactions that exist between the roots of inoculated plants and the major microbial communities involved in soil N dynamics. In particular, the changes in root growth, architecture and functioning induced by inoculation can modulate the availability of N in the rhizosphere, by enhancing plants-microbes competition for N and modifying rhizosphere environmental variables important for N-cycling microbial communities. This is the case for communities involved in processes such as nitrification, i.e. the oxidation of ammonium, NH_4_^+^, to NO_3_^− ^^23^. It is also the case for denitrification, i.e. the oxidation of NO_3_^−^ and nitrite, NO_2_^−^, into gaseous N forms^24^. Specifically, an increased root development and activity can increase the competition by plants for NH_4_^+^ and NO_3_^−^, thus decreasing the N substrate for nitrifiers and denitrifiers, respectively. In addition, the increased amount of easily available C released by roots as a result of phytostimulation by PGPRs can enhance mineralization and denitrification by increasing the abundance and growth of heterotrophic microorganisms in the rhizosphere^25, 26^. Oxygen availability in soil can also be affected by increased root growth. On one hand, oxygen can be lowered as a result of increased respiration by roots and root-associated microorganisms^27^. On the other hand, modified plant transpiration can affect soil water depletion and thus the diffusion of oxygen into soil^12^. The extent of these processes might differentially affect nitrification and denitrification, since these processes are favoured by aerobic and anoxic conditions, respectively^28^.

Although the relationships between host plants and PGPRs are well documented^2, 19^, the subsequent effects of PGPR inoculation on N-cycling processes and microorganisms have been barely investigated. Previous studies reported either no inoculation effect^29–31^ or significant effect on the composition of the indigenous total bacterial communities^32^ and a stimulation of the activity of arbuscular mycorrhiza^33^. However, no information is available about PGPR-induced effects on rhizospheric N-cycling processes and microorganisms.

The objective of this study was to evaluate the effects of maize inoculation with the PGPR *A*. *lipoferum* CRT1 on soil N-cycling processes and microorganisms. For these reasons, a multi-site field experiment was set up to investigate these inoculation effects. The potential activities and abundances of nitrifiers (bacterial and archaeal ammonia oxidizers, AOB and AOA, respectively) and denitrifiers (*nirK*- and *nirS*-harbouring NO_2_^−^ reducers, and *nosZI* and *nosZII* N_2_O-reducers) were measured in the rhizosphere of inoculated and non-inoculated maize plants at three dates over two consecutive years, for four conventionally or organically managed maize sites at each sampling date. We also measured soil environmental conditions (moisture, mineral N concentrations, organic C and pH) at all dates, and non-potential denitrification rates (i.e. without C or N added) at one date. We assumed that (i) inoculation effect would be mostly negative on the activity and abundance of nitrifiers, given that maize inoculation by *A*. *lipoferum* CRT1 is expected to increase the competition between plant roots and nitrifiers for NH_4_^+^. However, maize inoculation is expected to increase the competition between plant roots and denitrifiers for NO_3_^−^ and at the same time increase the input of root exudates-C usable by denitrifiers. We thus assumed that (ii) inoculation effect may be negative or positive on denitrification and denitrifier abundance according to the type of resource (i.e. N or C) mostly limiting denitrifiers in soil, which would make the inoculation effect on denitrifiers site-specific. Structural equation modelling, SEM, was used to identify the main drivers of the responses of (de)nitrifiers to inoculation.

## Results

### NO_3_^−^ and NH_4_^+^ uptake capacities of the maize cultivar studied

N uptake rate by the maize cultivar *cv*. *Seiddi*, was 260% higher at 1000 than 300 µM total mineral N (Supplementary Fig. S1). At 300 µM, NO_3_^−^ and NH_4_^+^ uptake rates were 0.019 and 0.006 mg h^−1^ g^−1^ root, respectively. In contrast, at 1000 µM these rates were 0.050 and 0.010 mg N h^−1^ g^−1^ root respectively (Supplementary Fig. S1). The ratios of NO_3_^−^ to NH_4_^+^ uptake rates were thus 3.1 at 300 µM N, and 4.9 at 1000 µM N (Supplementary Fig. S1).

### Effects of inoculation on potential nitrification and denitrification activities

In the results presented thereafter, abbreviations refer to the two years of experimentation (Y1, Y2) and two maize physiological stages corresponding to the sampling dates (6 leaves - 6 L, and 12 leaves - 12 L). At each date, four sites (Slope - S, Valley - V, Plateau - P and Valley Organically fertilized - VO), and different fertilization treatments (nf - not fertilized, f - fertilized, fs - fertilized at sowing, f/2 - reduced fertilization) were studied.

ANOVA with inoculation, site, and sampling date as fixed effects and the block factor as a random effect, was applied to identify the factors influencing potential nitrification activity, PNA. ANOVA results showed a significant main effect for date and site (p ≤ 0.0004) and a significant interaction effect between date, site and inoculation (p ≤ 0.035) (Table 1). This indicates that (i) PNA levels significantly differed on average between dates and between sites, and (ii) PNA was significantly influenced by inoculation but with effects varying across sampling dates and sites. Actually, PNA was on average lowest at Y1-12L (from 0.69 to 1.22 µg N g^−1^ h^−1^ according to sites and treatments) and highest at Y2-6L (from 0.67 to 1.82 µg N g^−1^ h^−1^) (Supplementary Fig. S2). In addition, PNA was significantly influenced by inoculation but this effect was not consistent, neither across sampling dates nor across sites (Table 2). For example, PNA was significantly increased by inoculation in S-fs plots at Y1-6L (p < 0.05) and in S-f plots (p < 0.01) at Y2-6L; but it was reduced in P-nf plots at Y1-6L (p < 0.05) and in V-nf plots at Y2-6L (p < 0.05). PNA was not significantly affected on the other plots (Table 2).

**Table 1:** Overall effects of maize inoculation by *Azospirillum lipoferum* CRT1 on potential nitrification activity, PNA, and potential denitrification activity, PDA, identified using ANOVA with inoculation, site, and sampling date as fixed effects and the factor block as a random effect.

	PNA p value	PDA p value
Sampling date	**0.0004**	<**0.0001**
Inoculation	0.527	**0.006**
Site	<**0.0001**	<**0.0001**
Block	**0.001**	<**0.0001**
Sampling date x Site	<**0.0001**	**0.0005**
Inoculation × Site	0.798	0.051
Sampling date × Inoculation	<**0.0001**	0.755
Sampling date × Inoculation × Site	**0.035**	0.927

**Table 2:** Effects of maize inoculation by *Azospirillum lipoferum* CRT1 on potential nitrification activity, PNA, and potential denitrification activity, PDA, at 6-leaves stage, year 1 (Y1-6L), 12-leaves stage, year 1 (Y1-12L), and 6-leaves stage, year 2 (Y2-6L).

Sampling year/season	Site	Fertilization	PNA (block-corrected)		PDA (block-corrected)	
Inoculation			NI	I	NI	I
Y1-6L	S	nf	−0.17 ± 0.13	−0.10 ± 0.07	−0.27 ± 0.28	0.32 ± 0.18
		fs	**−0.10 ± 0.08**	0.36 ± 0.12*	**−0.54 ± 0.22**	0.49 ± 0.20**
	V	nf	−0.35 ± 0.23	0.35 ± 0.23	**−0.34 ± 0.10**	0.34 ± 0.10***
	P	nf	**0.04 ± 0.02**	−0.04 ± 0.02*	0.06 ± 0.06	−0.06 ± 0.06
Y1-12L	S	nf	−0.13 ± 0.12	−0.17 ± 0.07	−0.28 ± 0.20	0.04 ± 0.22
		f	0.06 ± 0.06	0.08 ± 0.14	0.01 ± 0.14	0.20 ± 0.36
		fs	0.02 ± 0.07	0.14 ± 0.14	**−0.33 ± 0.15**	0.36 ± 0.15*
	V	nf	−0.16 ± 0.04	0.14 ± 0.23	−0.17 ± 0.08	0.27 ± 0.31
		f	−0.08 ± 0.17	0.10 ± 0.13	**−0.64 ± 0.28**	0.55 ± 0.36*
	P	nf	−0.10 ± 0.03	−0.01 ± 0.07	0.09 ± 0.10	0.16 ± 0.13
		f	0.01 ± 0.03	0.10 ± 0.09	−0.03 ± 0.13	−0.23 ± 0.08
Y2-6L	S	nf	0.00 ± 0.08	−0.06 ± 0.04	0.02 ± 0.30	0.35 ± 0.26
		f/2	0.09 ± 0.10	0.16 ± 0.09	**−0.50 ± 0.20**	0.78 ± 0.33*
		f	**−0.36 ± 0.07**	0.18 ± 0.18*	**−1.16 ± 0.26**	0.50 ± 0.42**
	V	nf	**0.31 ± 0.12**	−0.39 ± 0.25*	−0.06 ± 0.33	0.60 ± 0.42
		f	0.38 ± 0.16	−0.29 ± 0.37	−0.75 ± 0.28	0.20 ± 0.44
	VO	org	0.02 ± 0.04	−0.02 ± 0.04	**0.04 ± 0.02**	−0.04 ± 0.02*
	P	nf	−0.01 ± 0.10	0.06 ± 0.12	0.21 ± 0.13	0.08 ± 0.13
		f	−0.08 ± 0.08	0.03 ± 0.15	−0.10 ± 0.11	−0.19 ± 0.12

Values are corrected for block effects. Means ± standard errors (n = 5) are presented. S, V and P refer to the Slope, Valley and Plateau sites, respectively. nf, f, fs and f/2 refer to not fertilized, fertilized, fertilized at sowing, and reduced fertilization, respectively. VO-org refers to Valley site organically fertilized.

Regarding potential denitrification activity, PDA, that represents the potential (N_2_O + N_2_) production, significant main effects of sampling date, site and inoculation were observed (Table 1, p ≤ 0.0061). The inoculation X date and the inoculation X site X date interaction effects were not significant (p ≥ 0.75) and the inoculation X site interaction effect was nearly significant (p = 0.0511) (Table 1). Actually PDA tended to be lowest at Y1-12L (from 0.90 to 3.06 µg N g^−1^ h^−1^ according to sites and treatments) and highest at Y2-6L (from 0.98 to 5.01 µg N g^−1^ h^−1^) (Supplementary Fig. S2). In addition, PDA was significantly influenced by inoculation and the effects were largely consistent across sampling dates, but differed between sites (Table 2). PDA increased in response to inoculation in S-f or S-fs plots at the three sampling dates (Table 2). PDA also increased in response to inoculation in V plots at Y1-6L (p < 0.001) and V-f plots at Y1-12L (p < 0.05). However, it slightly decreased in VO plots at Y2-6L (Table 2). No significant effect of inoculation on PDA was observed in the other plots.

### Effects of inoculation on nitrifier and nitrite-reducer abundances

The abundances of AOB and AOA were of the same order of magnitude (i.e. typically from 1 to 5 × 10^7^ *amoA* copies g^−1^ dry soil for both groups, Supplementary Fig. S3) and were significantly correlated between each other (p < 0.001) only at Y2-6L sampling date. AOA abundances were significantly influenced by inoculation only at Y1-6L sampling date in P-nf. In contrast, AOB abundances were not significantly affected by inoculation at any time (data not shown).

The abundances of *nirK*- and *nirS*-harbouring denitrifiers are reported in Supplementary Fig. S4. The abundances of both groups were similar in S and VO soils, whereas the abundance of *nirK*-denitrifiers was around 10-fold higher (respectively 2–3 fold-lower) than the abundance of *nirS*-denitrifiers in P (respectively V) soil (Supplementary Fig. S4). The abundances of the two nitrite reducer groups were correlated between each other at Y1-6L and at Y2-6L sampling dates (p < 0.05) but not at Y1-12L. The abundance of *nirK*-denitrifiers was influenced by inoculation at Y1-12L in S-f plots and at Y2-6L in P-nf (p < 0.05), whereas *nirS*-denitrifier abundance was significantly affected by inoculation at Y1-6L and Y1-12L in S-fs plots, and at Y2-6L in S-f/2 plots (p < 0.05). No significant inoculation effect was observed in the other plots.

### Relationship between the inoculation effects on potential (de)nitrification and on (de)nitrifier abundance

**Figure 1:**
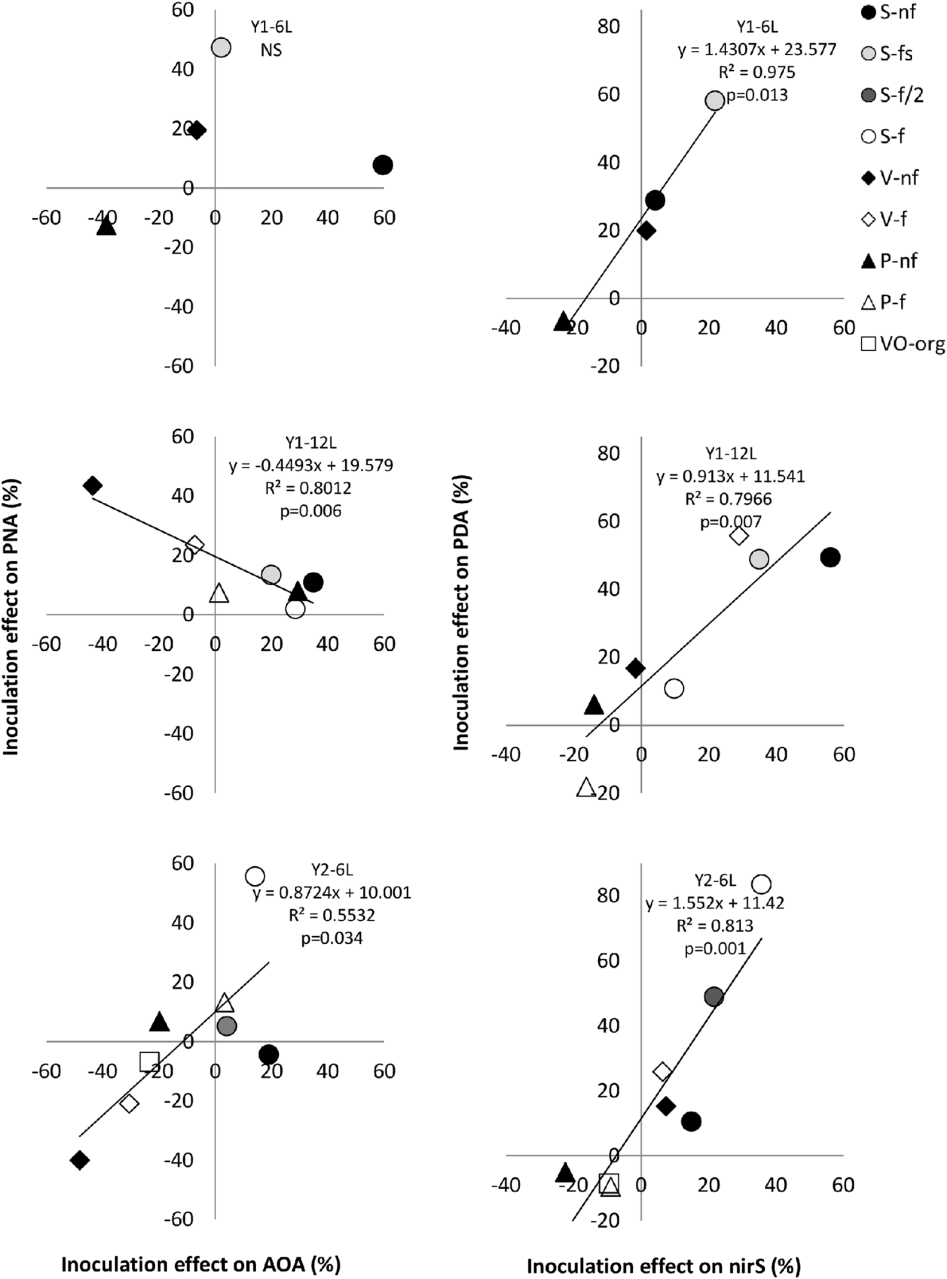
Relationship between the inoculation effects on (de)nitrifiers activity and on (de)nitrifiers abundance. Relationship between (left) the inoculation effects on potential nitrification activity, PNA, and on the abundance of ammonia-oxidizing archaea, AOA; and (right) the inoculation effects on potential denitrification activity, PDA, and on the abundance of *nirS*-denitrifiers. For each variable, the effect of inoculation was expressed as: % inoculation effect = [(I)/NI − 1] * 100. Each point corresponds to the mean value observed for n = 5 plot pairs (i.e. with and without inoculation). Relationships are presented for (Top) the 6-leaves stage during year 1, Y1-6L, (Middle) the 12-leaves stage during year 1, Y1-12L, and (Bottom) the 6-leaves stage during year 2, Y2-6L. V, S and P refer to the Valley, Slope and Plateau sites, respectively; nf, f, fs and f/2 refer to not fertilized, fertilized, fertilized at sowing and reduced fertilization, respectively. VO-org refers to Valley site organically-fertilized.

The inoculation effect on PNA (i.e. = (PNA_I_/PNA_NI_ − 1) * 100, see Methods section) was not correlated to inoculation effect on AOB abundance whatever the sampling date (data not shown; p > 0.40 at all dates). A significant positive correlation (R^2^ = 0.55, p = 0.034), a significant negative correlation (R^2^ = 0.80, p = 0.006) or a lack of correlation (p = 0.86) between the inoculation effects on PNA and on AOA abundance were observed at Y2-6L, Y1-12L and Y1-6L, respectively (Fig. 1, left). A high variability in the nitrifier response to inoculation was observed for individual sites over time: for instance, PNA in V-nf plots increased by 19% and 43% at Y1-6L and Y1-12L respectively, and decreased by 40% at Y2-6L. Similarly, AOA abundance in P-nf plots decreased by 39% and 20% at Y1-6L and Y2-6L respectively, but increased by 29% at Y1-12L (Fig. 1, left).

For each site and for each sampling date, the inoculation effects on PDA and on *nirS*-denitrifier abundance were correlated (Fig. 1, right). This was explained by the positive effect of inoculation on both PDA and *nirS*-denitrifier abundance in S site (from +10% to +84% and from +4% to 56% for PDA and *nirS*, respectively) and most often in V site (from +15% to +56%, and from −2% to 29%), and the negative inoculation effects in P site (from −18% to −7% for PDA, except for P-f at Y1-12L; and from −23% to −9% for *nirS*) and in VO site (−9% for both PDA and *nirS*). The inoculation effect on PDA was not correlated to the effect on the abundance of neither *nirK-*denitrifiers nor the sum of *nirK-* plus *nirS-*denitrifiers for two of the three sampling dates (data not shown).

### Semi-potential denitrification activities

Semi-potential denitrification activities, SPDA (i.e. when C, SPDA_C−N+_, or N, SPDA_C+N−_, sources were not added) were measured at the Y2-6L sampling date in order to elucidate the main resource(s) limiting denitrification among sites. At each site, endogenous soil organic C was more limiting for denitrification than NO_3_^−^ (Supplementary Fig. S5), but the extent of this limitation varied across sites. Denitrification was strongly limited by soil organic C in S and V sites, SPDA_C−N+_ being reduced by 76% to 67% as compared to PDA in all the plots in these sites (Supplementary Fig. S5). Denitrification was less limited by soil organic C in VO site (SPDA_C−N+_ reduced by 46% as compared to PDA) and P site (SPDA_C−N+_ reduced by 37% as compared to PDA). When N was not added, SPDA_C+N−_ was only slightly reduced as compared to PDA (by 12.6% for VO plots, and up to 35% for S-f/2).

### Relationship between the inoculation effect on PDA and the limitation of denitrifiers by organic C

**Figure 2:**
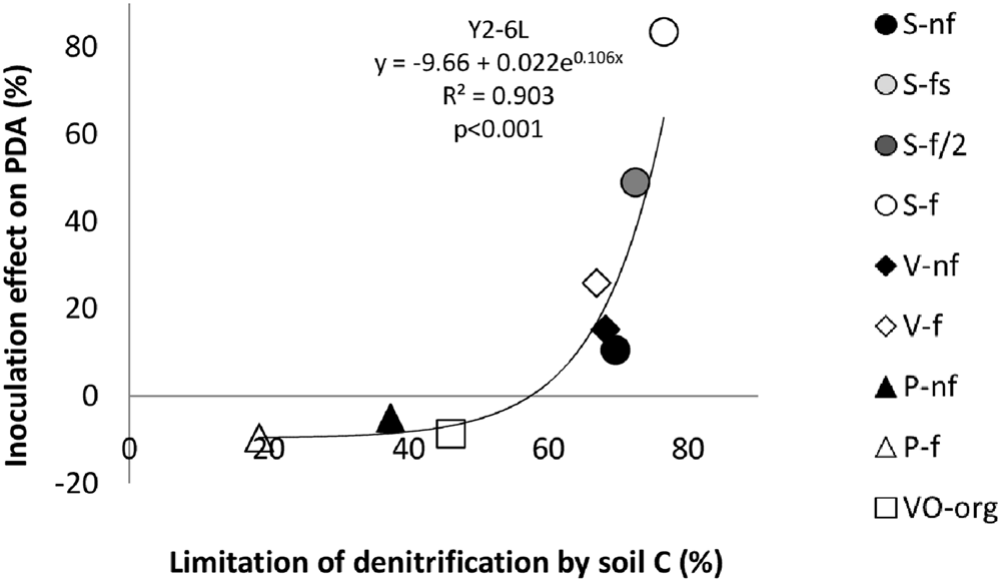
Relationship between the effect of inoculation on PDA and the level of limitation of denitrification by endogenous soil C_org_. Relationship between the effect of inoculation on potential denitrification activity, PDA, and the level of limitation of denitrification by endogenous soil C_org_ at the 6-leaves stage, year 2 (Y2-6L). C limitation was assessed from values of semi potential denitrification measured without C addition and PDA values. Symbols for sites and fertilization treatments are as in Fig. 1. Each point corresponds to the mean value observed for n = 5 plot pairs (i.e. with and without inoculation).

At Y2-6L, a positive and exponential relationship (R^2^ = 0.90, p < 0.001) was observed between the inoculation effect on PDA and the level of denitrifier limitation by soil organic C (Fig. 2), computed from values of semi potential denitrification measured without C addition as compared with PDA values (see Methods section). S soils, for which the limitation of denitrifiers by organic C was highest, showed the highest increases of PDA in response to inoculation (up to 84%), whereas VO and P soils characterized by a lower limitation of denitrifiers by organic C showed a slight reduction of PDA in response to inoculation (up to −9% for the site with the lowest C limitation).

### Relationships between the effect of inoculation on (de)nitrification, its effects on soil environmental variables and on (de)nitrifier abundances, and soil type

**Figure 3:**
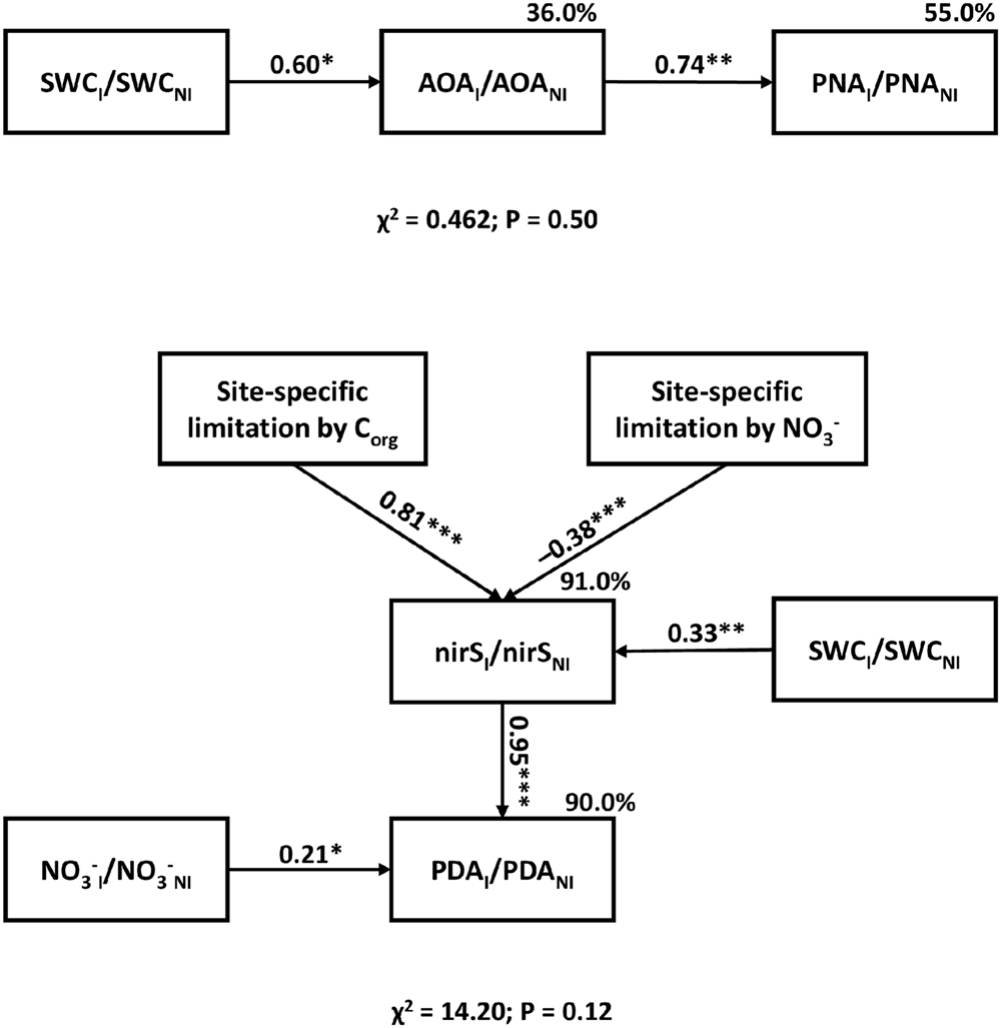
Structural equation models identifying probable causal effects of inoculation on (de)nitrification. Structural equation models identifying probable causal effects of inoculation on (Top) potential nitrification activity, PNA, and (Bottom) potential denitrification activity, PDA. The full models tested are presented in Fig. S8. For soil ammonium, NH_4_^+^, nitrate, NO_3_^−^, and water content, SWC, potential activities (PNA and PDA) and gene abundances (AOA and *nirS*-denitrifiers), the effect of inoculation (%) was computed as [(I)/NI − 1] * 100. The proxies of the site-specific levels of denitrification limitation by C and N were computed from PDA and semi-potential denitrification activity measured when only N or C, respectively, were added. Path coefficients (values near the arrows) correspond to the standardized coefficients based on the analysis of correlation matrices. Significant relationships (*P < 0.05; **P < 0.01; ***P < 0.001) are indicated with solid arrows. For activities and gene abundances, the percentages of variance explained by the model are indicated.

The best fitted models identified by structural equation modeling for the Y2-6L sampling date for identifying the main drivers of inoculation effect on PNA (χ^2^ = 2.55, P = 0.47) and PDA (χ^2^ = 14.20, P = 0.12) are reported in Fig. 3. The effect of inoculation on AOA abundance was affected (36% of the variance explained by the model) by inoculation-induced changes in SWC (p < 0.05, Fig. 3, Top). The inoculation effect on PNA at this date was linked (55% of the variance explained by the model) to inoculation-induced changes in AOA (p < 0.01) (Fig. 3, Top).

The effect of inoculation on the abundance of *nirS*-denitrifiers was strongly and positively linked to the site-specific level of denitrifier limitation by soil C and to the inoculation effect on SWC (p < 0.001 for both factors), and negatively linked to the site-specific levels of denitrifier limitation by soil NO_3_^−^ (p < 0.001, Fig. 3, Bottom) (91% of the variance of inoculation effect on abundance explained in total). In addition, the inoculation-induced changes in *nirS*-denitrifier abundance and in soil NO_3_^−^ concentration (p < 0.001 and p < 0.05, respectively) were positively related to the inoculation-induced changes in PDA (90% of the variance explained, Fig. 3, Bottom). When we included a connection between PNA and PDA, the resulting SEM models were not significant (data not shown).

### Relationship between the inoculation effects on the abundance of nitrite reducers and on the abundance of N_2_O-reducers

The abundances of *nosZI* and *nosZII* ranged from 3 × 10^5^ to 2 × 10^6^ and from 2 × 10^5^ to 1 × 10^7^copies g^−1^ dry soil, respectively (data not shown).The inoculation effect on the abundance of *nirS*-denitrifiers was significantly (R^2^ = 0.49, p = 0.0008) related to the inoculation effect on the total abundance of N_2_O reducers (i.e. the sum of *nosZI* and *nosZII*–harbouring denitrifiers) (Supplementary Fig. S6).

## Discussion

In the rhizosphere, interactions between plant and N-related processes and microorganisms are modulated by different factors. In particular, N limitation in soil and mineral N uptake by plants generate plant-microbes competition for NH_4_^+^ and NO_3_^− ^^34^, while the release of root C exudates favours the activity and growth of soil heterotrophic microorganisms^1^. Root exudation can also modulate soil nutrient availability by altering soil chemical and/or biological processes, influencing in turn the outcomes of resource competition between plants and microorganisms. Our results demonstrate that maize inoculation by the PGPR *A*. *lipoferum* CRT1 affects the outcome of interactions between plants and soil nitrifying and denitrifying communities.

### Determinants of the responses of nitrifier activity and abundance to inoculation

Nitrifiers are able to uptake soil NH_4_^+^ up to five times more rapidly than plants^35^ because they have a higher surface area-to-volume ratio than roots^36^, but roots acquire N more continuously throughout the plant life cycle, leading to a progressive N depletion in the rhizosphere^34^. Previous studies have reported lower PNA and ammonia-oxidizer abundance^37^ for plant species with a higher root development. This is relevant for cereal crop plants inoculated with PGPRs. In particular, Babić *et al*.^38^ observed decreased AOB (but not AOA) abundance as well as decreased soil NH_4_^+^ concentration in *Medicago sativa L*. pots inoculated with *Sinorhizobium meliloti*. Our hypothesis that increased plant-nitrifiers competition for NH_4_^+^ would play a key role for inoculation effect on nitrifiers obviously requires that NH_4_^+^ can be efficiently taken up by crop roots. However, the level of NH_4_^+^ uptake is known to vary with the soil NH_4_^+^-to-NO_3_^−^ ratio and with plant species or variety^39^.

Our results show that maize inoculation with *A*. *lipoferum* CRT1 resulted in a highly variable response of nitrifier activity (from −40% to +55%) and nitrifier abundance (from −50% to +60%), the response changing from one sampling date to another at a given site, and between sites and treatments at a given date. This idiosyncratic response of nitrifiers to inoculation thus cannot be simply related to the sole competition between nitrifiers and maize roots for NH_4_^+^. This is due to a less prominent role of NH_4_^+^ uptake by roots than anticipated and higher importance of other processes. Firstly, our results show that the maize *cv*. *Seiddi* largely preferred NO_3_^−^ over NH_4_^+^ at the two levels of total mineral N availability tested. During the N root uptake assays, we used a proportion of NH_4_^+^-to-total mineral N close to that observed in soil in the field (i.e. 10% as compared to values always lower than 9% for the different sites and treatments); this prominence of NO_3_^−^ over NH_4_^+^ is commonly observed for cropped soils^40^. It is thus very likely that maize *cv*. *Seiddi* plants were not competing effectively with nitrifiers for soil NH_4_^+^. Secondly, enhanced root exudation may stimulate total microbial activity and biomass and microbial demand for nutrients, which can be met by increased mineralization of organic matter from soil and root exudates^41, 42^. Inoculation may thus have not altered or even increased soil NH_4_^+^ availability due to increased organic matter mineralization^43^, thus not influencing or even alleviating the plant-nitrifiers competition for NH_4_^+^. Thirdly, SEM results showed that a key determinant of PNA response to inoculation at the third date was the inoculation effect on soil water content, SWC, which induced changes in AOA abundance (Fig. 3, Top). It is known that oxygen availability, which is influenced by SWC, modifies nitrification in soil^28^. Nitrification is generally maximal for SWC around 70% of water holding capacity and decreases if moisture becomes too low and too high due to drought stress and lack of oxygen, respectively^44^. The key role of soil moisture in explaining the inoculation effect on nitrifiers observed here is consistent with report from Sarig *et al*.^12^ who observed larger leaf area and greater evapotranspirational demand in *Sorghum bicolor* inoculated with *Azospirillum brasilense* with greater soil moisture depletion by plants, and from Grover *et al*.^45^.

The difference observed for the responses of the two groups of ammonia oxidizers to inoculation can be due to the different metabolims and ecophysiological traits of AOA and AOB. For example, AOA are often less responsive to environmental changes than AOB^46–48^. Reports of high AOA abundances in various ecosystems and in deeper soil layers strongly suggest that AOA are adapted to a broad range of growth conditions and might have a more versatile metabolism than AOB, AOA likely being able to grow mixotrophically^49^.

Overall, our results demonstrate that inoculation effect on nitrifier activity and abundance could not be explained by an increased plant-nitrifiers competition for soil NH_4_^+^. Actually, inoculation effect on nitrifiers was mostly linked to modifications of water balance and soil moisture. The resulting inoculation effect on soil moisture depends on processes acting at the entire soil-plant system level (including plant transpiration and soil evaporation rates), on precipitation regime and on key soil characteristics regarding water retention. This can explain the apparent idiosyncratic response of nitrifier activity and abundance to PGPR inoculation.

### Determinants of the response of denitrifier activity and abundance to inoculation

For a given site and treatment, the effects of maize inoculation on denitrifier activity (from −18% to +84% between treatments) and *nirS*-abundance (from −23% to +56%) were roughly consistent across sampling dates. Furthermore, the inoculation effect on the abundance of *nirS*-denitrifiers largely explained the inoculation effect on PDA, which hold across plant growth stages and for different years. Although *A*. *lipoferum* possesses the *nirS* gene and can denitrify^50^, the impact of inoculation on denitrifier abundance and PDA could not be explained by the direct effect of *A*. *lipoferum* addition on maize seeds that would then result in actively growing populations of this denitrifier in soil. Indeed, this strain could not be detected by quantitative PCR at the 6-leaves stage in any experimental fields (qPCR counts were always below the detection limit). Our results are consistent with studies reporting that PGPRs inoculated on cereal seeds stimulate root growth and modify plant metabolism at very early stages and generate lasting effects on the root system, but cannot compete efficiently with native microbial communities in soil and disappear quickly, typically after a few weeks^51^.

Changes in PDA could result from changes in the physiological activity of denitrifier cells (activity per cell), and/or changes in denitrifier abundance. Indeed, the synthesis of the key enzymes involved in denitrification is inducible^24^, and the activity and abundance of denitrifiers are thus not necessarily tightly coupled. However, changes in the abundance of *nirS*-denitrifying bacteria in response to inoculation appear as a key driver of inoculation-induced changes in PDA, which is clearly illustrated in our SEM results. Some studies found that *nirK*- rather than *nirS*-bacteria form the major part of soil denitrifiers^37, 52^ and are mainly responsible of denitrification in cropped soils^53, 54^. However, the relative importance of *nirS* and *nirK*– harbouring denitrifiers and a possible niche differentiation between these two groups are still debated^55, 56^. Overall, our results indicate that the inoculation effect on PDA was largely mediated by the build-up of the *nirS*-denitrifier community. Similarly, changes in the abundance of *nirS*-denitrifiers explained changes in PDA in different soils^57, 58^.

Interestingly, inoculation decreased PDA in P and VO soils for each of the three sampling dates, whereas it increased PDA in S and V soils. Contrasting responses of denitrification activity to the presence (or to a modified growth and activity) of a root system have been observed in other studies. Qian *et al*.^59^ reported lower denitrification rates due to the presence of maize roots as compared to unplanted soil, whereas Mahmood *et al*.^60^ and Philippot *et al*.^61^ reported denitrification rates stimulated by maize root system. Our working hypothesis was that the relative importance of the negative effect of an increased plant-denitrifiers competition for NO_3_^−^ and the positive effect of an increased root exudation would vary according to the level of denitrifier limitation by soil NO_3_^−^ and soil C availability. Our results support this, and show that the higher the limitation of denitrifiers by soil C availability, the higher the increase in the denitrifier abundance and activity in response to inoculation (C limitation reached 76% in S-f plots where the stimulation of PDA and *nirS* abundance reached +84% and +36%, respectively). Further, negative effect of inoculation on denitrifiers was observed at the lowest levels of denitrifier limitation by soil C (C limitation was only 19% in P-f site where both PDA and *nirS* abundance decreased by −9%). Fertilization weakly influenced the relationship between inoculation effect on PDA and C limitation level. Indeed the levels of denitrifier limitation by C remained higher than the levels of denitrifier limitation by NO_3_^−^ even in non-fertilized crop fields (Supplementary Fig. S5). Our results thus underline the key role of C availability for explaining soil denitrifier response, in accordance with those of Weier *et al*.^62^, Schaeffer *et al*.^63^ and Attard *et al*.^54^ who found that denitrification is mainly regulated by C availability in cropping systems. This finding was further confirmed by the SEM analysis which revealed a positive and highly significant relationship between the site-specific C limitation level and the inoculation effect on *nirS*-denitrifiers (Fig. 3, Bottom). Furthermore, a negative relationship between the site-specific N limitation level and the inoculation effect on *nirS* abundance was observed. We conclude that in sites with high C limitation levels (S and V, limitation >66%), inoculation with *A*. *lipoferum* CRT1 probably stimulated root C exudation and possibly oxygen consumption in the rhizosphere, which favored *nirS*-denitrifiers and increased denitrification. Conversely, in sites with low C limitation (P and VO, limitation <47%), the stimulating effect of inoculation on root C exudation was less critical for denitrifiers whereas the increased competition between roots and denitrifiers for NO_3_^−^ became prominent, thus resulting in slightly decreased *nirS*-denitrifier abundance and denitrification.

In addition to these two major processes, the SEM analysis also revealed a significant effect of the inoculation-induced changes in SWC on *nirS*-denitrifier abundance. Soil moisture is often reported as a key soil environmental variable explaining changes in denitrification in soil^64, 65^. For instance, comparing 16 global change treatments applied to grasslands, Barnard *et al*.^66^ attributed 60% of the variance observed for PDA to changes in soil moisture.

Overall, our results show that, according to the site-specific levels of denitrifier limitation by C and N, inoculation can either increase or decrease *nirS*-denitrifier abundance due to the balance between alleviated C limitation and increased NO_3_^−^ limitation. This interacts with modified SWC and determines changes in PDA (this model explaining 90% of the variance associated to inoculation effect on PDA). The level of denitrifier limitation by endogenous soil C was a good predictor of the inoculation effect on denitrifiers. However, from a practical point of view, it has to be noted that the level of denitrifier limitation by soil C was not significantly related to soil C content (not shown). The ratio of *nirS*-denitrifier abundance to microbial basal respiration was a much better descriptor of the level of denitrifier limitation by soil C (Supplementary Fig. S7). Microbial basal respiration is often used a proxy of labile C availability in soil^67^, and this ratio thus represents the number of denitrifiers that compete per unit of labile C in soil, which explains why it is a better proxy of denitrifier limitation by soil C than soil C content. This proxy could be used to predict soils in which maize inoculation may increase the potential denitrification, i.e. N losses through the production of N_2_O and N_2_. Additional investigations including laboratory measurements of gross and net N_2_O production and *in situ* quantification of nitrous oxide fluxes for inoculated and non inoculated maize fields are required to quantify the effect of inoculation on N_2_O and N_2_ losses, particularly for soils where denitrifiers are highly C-limited.

## Methods

### Experimental design, treatments and soil sampling

Three experimental sites located along a catena within the Isère-Porte-des-Alpes (IPA) territory, southeast of France, were monitored in two consecutive years: a site in the valley (V site; 45°37′N, 5°16′E), a site on the slope (S site; 45°56′N, 5°33′E), and a site on the plateau (P site; 45°28′N, 5°14′E). A fourth site, adjacent to V site and managed under organic farming (45°57′N, 5°34E) (VO), was included in the second year of experimentation. Soil type according to WRB (2006)^68^ and the main soil physico-chemical properties are reported for each site in Table S1. The experiment was set up as a randomized block design with 5 blocks and treatments randomly assigned to one plot in each block. Plot dimensions were: 12 m × 9.6 m for S and V sites, 12 m × 6.4 m for P site, and 25 m × 4.8 m for VO site.

Maize (*Zea mays*, cv. *Seiddi*) seeds were inoculated with *A*. *lipoferum* CRT1 previously isolated from the rhizosphere of field-grown maize in France^69^. We targeted an inoculum load of 10^6^ colony forming units added per inoculated seed, I, coated in a commercial peat-based Azo-Green^TM^ formulation (Agrauxine, Beaucouzé, France). Coated but non-inoculated seeds, NI, were used as controls. According to the local agronomic practices, in 2014 seeds were sown on 18^th^ April in site S (98,000 seeds ha^−1^) and on 23^rd^ April in sites V and P (90,000 and 89,000 seeds ha^−1^, respectively). In 2015, sowing occurred on 30^th^ April in V and VO sites (98,000 seeds ha^−1^) and on 11^st^ May in V and P sites (95,000 and 89,000 seeds ha^−1^, respectively).

Each year, non-fertilized and fertilized plots were included in the experimentation in the sites V, S and P. In 2014, in each site 5 plots were not fertilized (i.e. V-nf, S-nf and P-nf plots) while mineral fertilizer (NH_4_NO_3_) was applied in 5 other plots at a rate of 120 kg N ha^−1^ (V-f and S-f plots) and 180 kg N ha^−1^ (P-f plots). The amounts of mineral fertilizer were chosen to be close to optimal N availability, taking into account the N fertilizer recovery efficiency by maize in the previous year. Another fertilization treatment was also included in S site, which consisted in the application of 60 kg N ha^−1^ at sowing (S-fs plots). This led to a total of 70 plots in 2014, i.e. 35 pairs of NI-I plots. In 2015, mineral fertilizer was applied at a rate of 60 kg N ha^−1^ (V-f plots), 80 kg N ha^−1^ (S-f plots) and 120 kg N ha^−1^ (P-f plots). In S, a reduced fertilization treatment was also used (30 kg N ha^−1^, S-f/2 plots), while in the VO site 10 plots received feather meal as organic fertilizer at a rate of 120 kg N ha^−1^ (VO plots). This led to a total of 80 plots for the second year, i.e. 40 pairs of NI-I plots.

In 2014, rhizosphere soil samples were collected at the 6-leaves stage (Y1-6L) on 25^th^ May (site S) and 26^th^ May (sites V and P), and at the 12-leaves stage (Y1-12L) on 8^th^ July (S and V) and 9^th^ July (P). In 2015, rhizosphere soil samplings were done on 27^th^ May (sites V and VO), 5^th^ June (site S) and 8^th^ June (site P) at the 6-leaves stage (Y2-6L). Six individual plants were randomly selected from each plot and removed using a spade to excavate the root system. Rhizosphere soil was collected by gently shaking the roots. Soil retrieved from the 6 plants was pooled, sieved using 2-mm mesh size and stored at +4 °C a few days before activity measurements. A sub-sample was frozen at −20 °C before DNA extraction.

For each fresh soil sample, a ~10 g subsample was weighed and dried at 105 °C during 24 h to determine gravimetric soil water content (SWC). Mineral N concentration was measured using 5 g equivalent dry weight soil after extraction with 20 mL of 2 mol L^−1^ KCl. The extraction solution was shaken at 10 °C for 1 h at 140 rpm, filtered at 0.2 µm and frozen at −20 °C until measurements of NO_3_^−^, NO_2_^−^ and NH_4_^+^ concentrations were made using an ion chromatograph (DX120 Dionex, Salt Lake City, USA) equipped with a 4 × 250 mm column (IonPac AS9 HC). Soil pH was determined on a soil:water mixture (1:5) using a pH analyzer (WTW InoLab multi 9420, Weilheim, Germany).

### Nitrification and denitrification assays

Potential nitrification activity (PNA) was measured using the method described by Dassonville *et al*.^70^. Briefly, samples of fresh soil (3 g dry weight equivalent) were incubated for 10 h with 30 ml of (NH_4_)_2_SO_4_ (1.25 mg N L^−1^) using continuous shaking (180 rpm, 28 °C). Subsamples (1 ml) were collected at 2 h, 4 h, 6 h, 8 h and 10 h, filtered (0.20 µm pore size) and stored at −20 °C. The NO_3_^−^ and NO_2_^−^ concentrations were analysed using an ion chromatograph (DX120, Dionex).

Potential denitrification activity (PDA) was measured in fresh soil according to Patra *et al*.^71^, using fresh soil samples (10 g dry weight equivalent soil). PDA was determined as the linear rate of production of N_2_O during short-term (8 h) incubation under anaerobic conditions using a gas chromatograph (µGC R3000, Santa Clara, CA, USA). Acetylene gas (C_2_H_2_) was used to inhibit nitrous oxide reductase activity and avoid N_2_ production. Glucose (0.5 mg C g^−1^ dry soil), glutamic acid (0.5 mg C g^−1^ dry soil) and KNO_3_ (200 µg NO_3_^−^ N g^−1^ dry soil) were added to the soil samples and the soil moisture was brought to 100% water holding capacity.

In addition, semi-potential denitrification activities when C was not added (SPDA_C−N+_) or when N was not added (SPDA_C+N−_) were measured for all the Y2-6L soil samples. In this case, denitrification depends on soil endogenous C and N supply, respectively. Activity measurements were performed on the AME platform (Microbial Ecology UMR5557, Lyon).

### Soil basal respiration

Soil basal respiration, i.e. the amount of endogenous soil C mineralized by the native soil community, was measured using the MicroResp^TM^ method, which consists in a 96-deep-well microplate filled with soil (40% of WHC, corresponding to 0.30 g dry weight soil per wells) and added (25 µl per deep-well) with water only. The plates were then sealed to a colorimetric CO_2_-trap microplate and incubated in the dark at lab temperature (23 °C±2) for 6 hrs^72^. The humidity was adjusted (MilliQ water) (40% of WHC corresponding to optimal edaphic conditions for microbial respiration) and soils were pre-incubated for one week at 23 °C (±2) in dark^73^. Absorbance was measured at 570 nm (Biotek EL-800 spectrophotometer). A calibration curve of absorbance *versus* headspace equilibrium CO_2_ concentration (measured by gas chromatography) was fitted to a regression model, which was used to compute the amounts of released CO_2_. The results were expressed in µg C-CO_2_ g^−1^ soil h^−1^ ^73^.

### Quantification of nitrifier and denitrifier abundances

DNA was extracted from 0.5 g of soil samples using the FASTDNA SPIN Kit for Soil (BIO 101 Systems; Qbiogene, Carlsbad, CA, USA). DNA concentrations were determined using a Qubit® 2.0 fluorometer with Quant-iT^TM^ dsDNA broad range (BR) Assay Kit (Invitrogen, France).

The abundances of ammonia-oxidizing bacteria, AOB, and ammonia-oxidizing archaea, AOA, were measured by quantitative PCR targeting the *amoA* functional gene encoding for ammonia monooxygenase which is specific of these groups. Amplification was performed as previously reported for AOA^74^ and AOB^75^. The abundance of denitrifiers was measured by quantitative PCR targeting the *nirK* or *nirS* genes (encoding the copper and *cd*_*1*_ nitrite reductases, respectively). Amplification was performed as previously reported for *nirK* ^76^ and *nirS* ^77^. Furthermore, *nosZI* and *nosZII* genes were targeted to quantify the abundances of N_2_O-reducing bacteria. Amplification was performed as previously reported for *nosZI* ^76^ and *nosZII* ^78^.

Standards were generated from PCR products that had been obtained from soil DNA extracts, gel purified, and quantified by fluorimetry. This minimises possible bias linked to the use of one or a few genes as standards for quantification of the size of complex communities^79^. Standards were diluted to give a concentration range from 0 to 10^8^ gene copies μl^−1^. Possible inhibitory effects of co-extracted humic compounds in soil extracts were checked by dilution series, but no inhibition was observed. The average real-time PCR efficiency for each of these genes was 94%, 99.8%, 95%, 99.3%, 75% and 87% for *amoA* AOA, *amoA* AOB, *nirK*, *nirS*, *nosZI* and *nosZII*, respectively.

### Measurements of NO_3_^−^ and NH_4_^+^ uptake rates by roots of maize cv. Seiddi

Uptake rates of NO_3_^−^ and NH_4_^+^ by roots of the maize cultivar Seddi were measured as described by Maire *et al*.^80^ with slights modifications. Maize seeds of *cv*. *Seiddi* (weight range 0.361–0.378 g) were sown in pots (11.3 × 11.3 × 21.5 cm) containing coarse sand (<4 mm). Plants were grown until 6-leaves stage in a growth chamber (photoperiod 16 h, temperature 19 °C night and 26 °C day, relative humidity 65%, PPFD: 350 µmol m^−1^s^−1^) and watered every two days. Once per week nutrient solution was added as described in Castle and Randall^81^. The entire root system was rinsed and immersed in pots containing the same nutrient solutions where NH_4_NO_3_ was replaced by KNO_3_ and (NH_4_)_2_SO_4_. Uptake kinetics were characterised using two total mineral N concentrations (300 and 1000 µM) and a NO_3_^−^:NH_4_^+^ ratio of 9:1 close to that observed in the soils studied (this ratio being always higher than 11:1 for the different sites and treatments studied). Five replicates per treatment were used. One mL aliquots were sampled after 0, 25, 50, 75 and 100 min of incubation, filtered (0.20 µm pore size) and stored at −20 °C. The NH_4_^+^, NO_3_^−^ and NO_2_^−^ concentrations were analysed using an ion chromatograph (DX120, Dionex). The root system of each plant was dried at 105 °C for two days. Uptake rates were expressed as mg N-NO_3_^−^ or N-NH_4_^+^ h^−1^ g^−1^ root dry mass.

### Statistical analyses

For each sampling date, significant effects of inoculation on microbial activities and abundances were identified using three-way ANOVA with inoculation, site and block as factors (JMP Pro 12, SAS Institute, Cary, North Carolina, USA). Two-way ANOVA with inoculation and block as factors were performed when no interactions effects with site were detected. The factors inoculation and site were treated as fixed effects and the factor block as a random effect. Where necessary, data were log-transformed to ensure conformity with the assumptions of normality and homogeneities of variances. The block effect was significant for activities and genes abundance at each sampling date. Residual values corrected for block effects were thus calculated and used to investigate significant effects of inoculation using paired t-test.

For each variable and each pair of NI-I plots at each date, the effect of inoculation was expressed as: % inoculation effect = [(I)/NI − 1] *100. Correlations were carried out using JMP Pro 12 to investigate the relationships between the effects of inoculation on PNA or PDA and (de)nitrifier abundances.

Furthermore, we used the comparison of SPDA to PDA to compute a proxy of the limitation of denitrifier activity by endogenous soil C_org_ as follows: (1 − SPDA_(C−N+)_/PDA)  * 100. Correlations were performed to investigate the relationships between denitrification limitation by C_org_ and inoculation effect on PDA.

We used structural equation modelling (SEM)^82^ to explore the causal links between the inoculation effects on microbial activities, microbial abundances and soil environmental parameters, using the software Amos18 (Amos Development Corporation Crawfordville, Florida, USA). In SEM, a χ^2^ test is used to determine whether the covariance structure implied by the model adequately fits the actual covariance structure of the data. A non-significant χ^2^ test (P > 0.05) indicates adequate model fit. In addition, paths (i.e. casual links between variables identified in the model) are considered significant if their P value was < 0.05. Paths coefficients (values indicated on the arrows) indicate by how many standard deviations the response variable would change if the driving variable were changed by 1 standard deviation. Theoretical structural equation models, identifying probable causal effects of inoculation on PNA and PDA are given in Fig. S8.

## Electronic supplementary material

Supplementary Material
