# Supplementary material for: Soil C and N statuses determine the effect of maize inoculation by plant growth-promoting rhizobacteria on nitrifying and denitrifying communities
Source: Sci Rep. 2017 Aug 21;7:8411. doi: 10.1038/s41598-017-08589-4 (PMC5566440; doi:10.1038/s41598-017-08589-4)

**Supplementary Material**

**Soil C and N statuses determine the effect of maize inoculation by plant growth-promoting rhizobacteria on nitrifying and denitrifying communities**

by Alessandro Florio, Thomas Pommier, Jonathan Gervaix, Annette Bérard & Xavier Le Roux

1 Supplementary Table S1

8 Supplementary Figures S1, S2, S3, S4, S5, S6, S7 and S8

**Table S1**. Soil types and physico-chemical properties of the soils investigated.

|  | **Slope soil (S)** | **Valley soil (V)** | **Valley soil-organically fertilized Bio (VO)** | **Plateau soil (P)** |
| --- | --- | --- | --- | --- |
| **Soil type^a^** | Fulvic Cambisol | Calcisol (siltic) | Calcisol (siltic) | Luvisol (skeletic) |
| **Clay (%)** | 34.7 | 10.3 | 10.2 | 14.2 |
| **Sand (%)** | 26.9 | 15.6 | 27.4 | 42.9 |
| **Silt (%)** | 38.3 | 74.1 | 62.5 | 42.9 |
| **pH (H_2_O)** | 7.11 | 7.93 | 8.09 | 7.09 |
| **C_org_ (g C kg^-1^)** | 31.6 | 25.9 | 20.0 | 21.5 |
| **Total N (g N kg ^-1^)** | 3.4 | 3.1 | 2.1 | 1.6 |
| **Olsen P (g kg^-1^)** | 0.153 | 0.132 | 0.134 | 0.171 |
| **K (g kg^-1^)** | 0.181 | 0.135 | 0.145 | 0.201 |
| **Ca (g kg^-1^)** | 5.950 | 10.110 | 11.940 | 2.940 |
| **Mg (g kg^-1^)** | 0.136 | 0.048 | 0.088 | 0.066 |

a: according to World Reference Base for Soil Resource (2006).


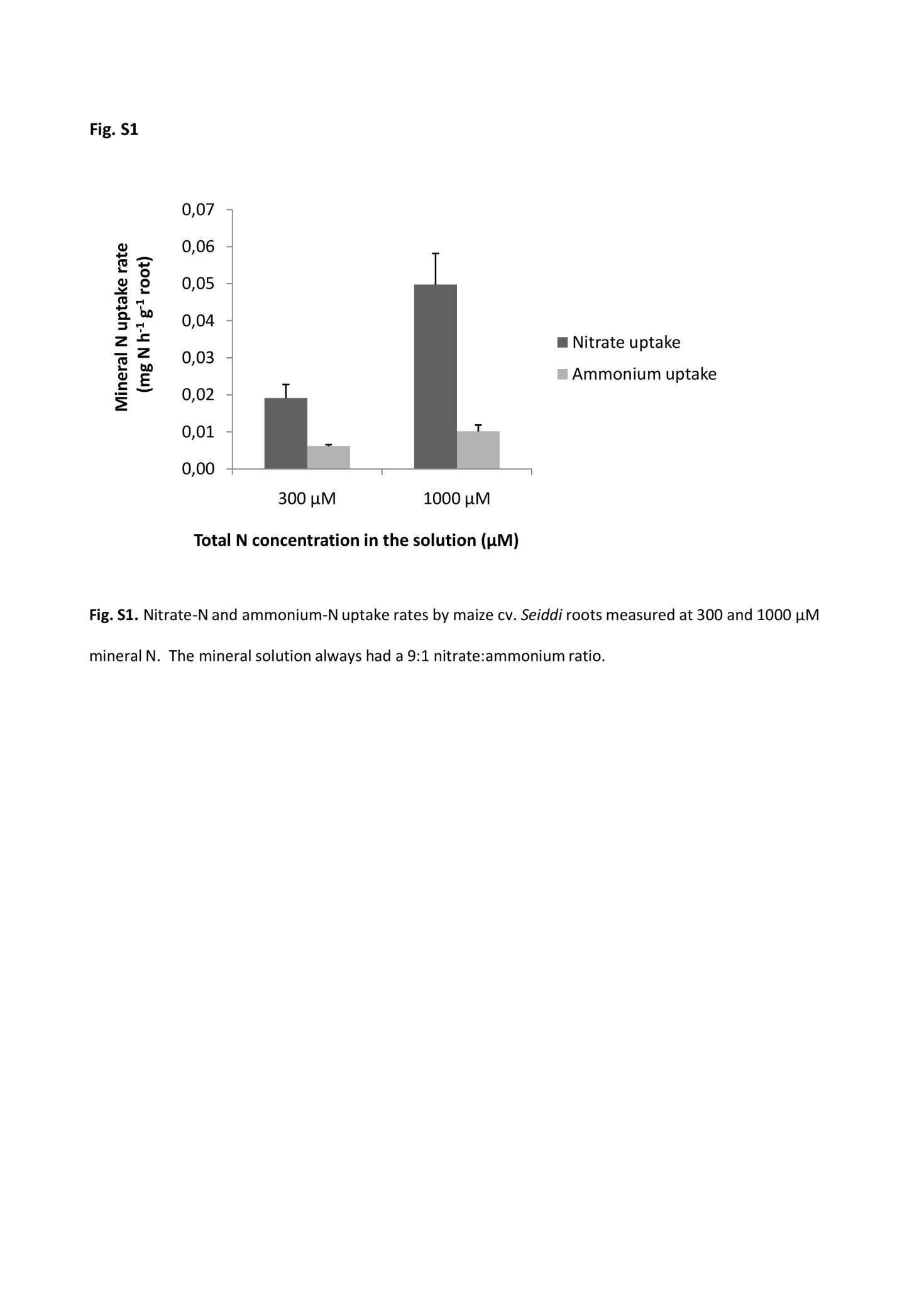

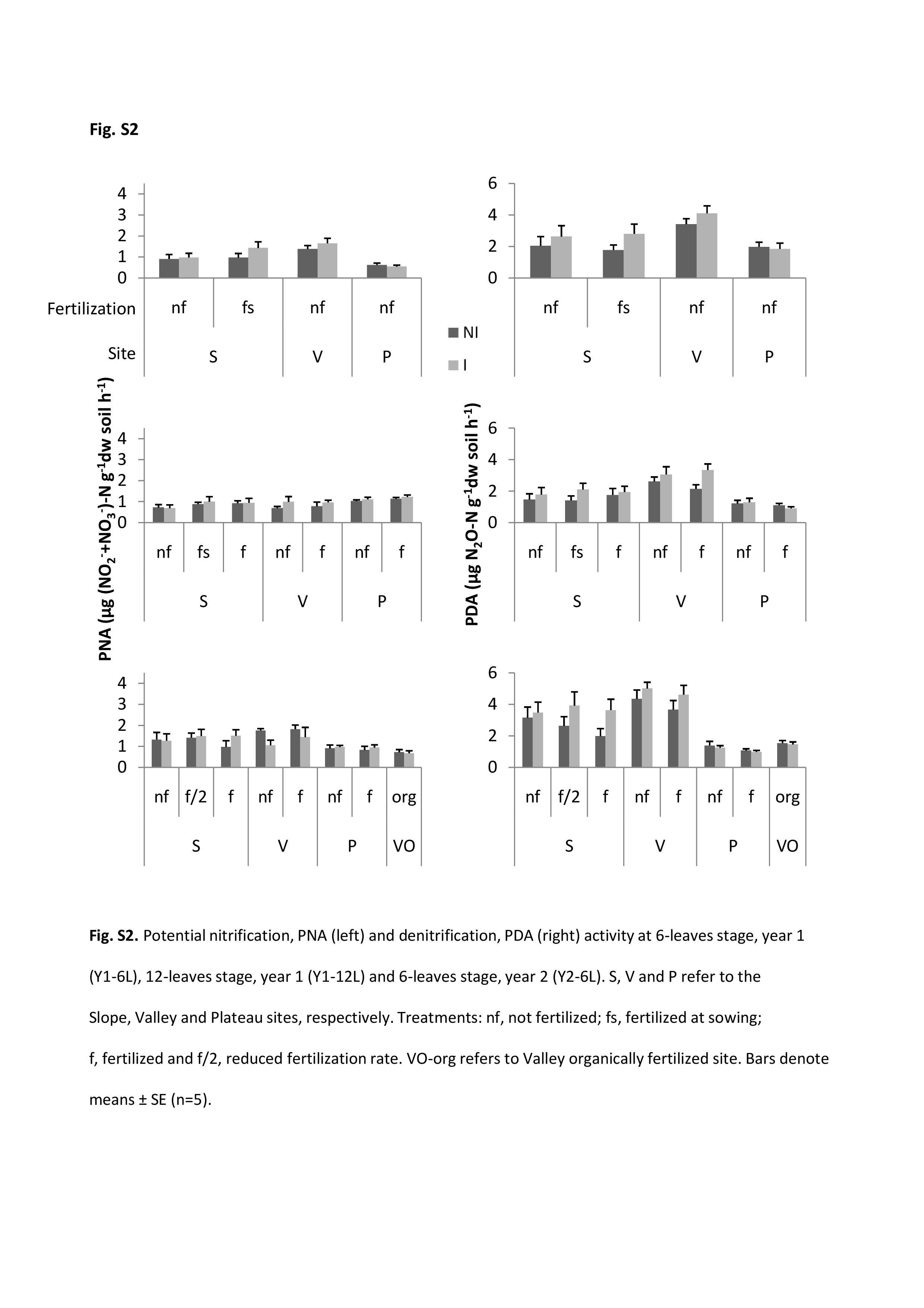

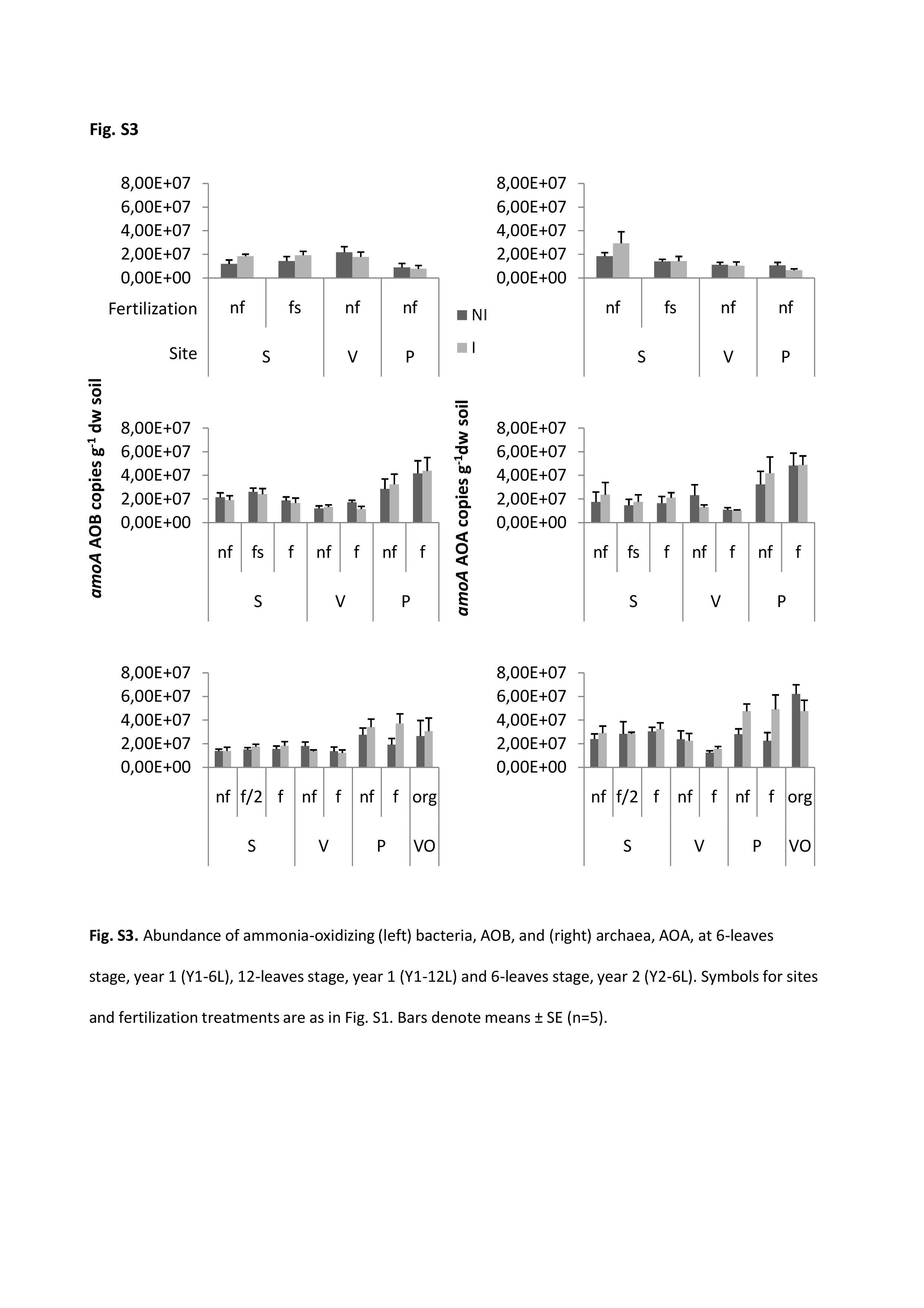

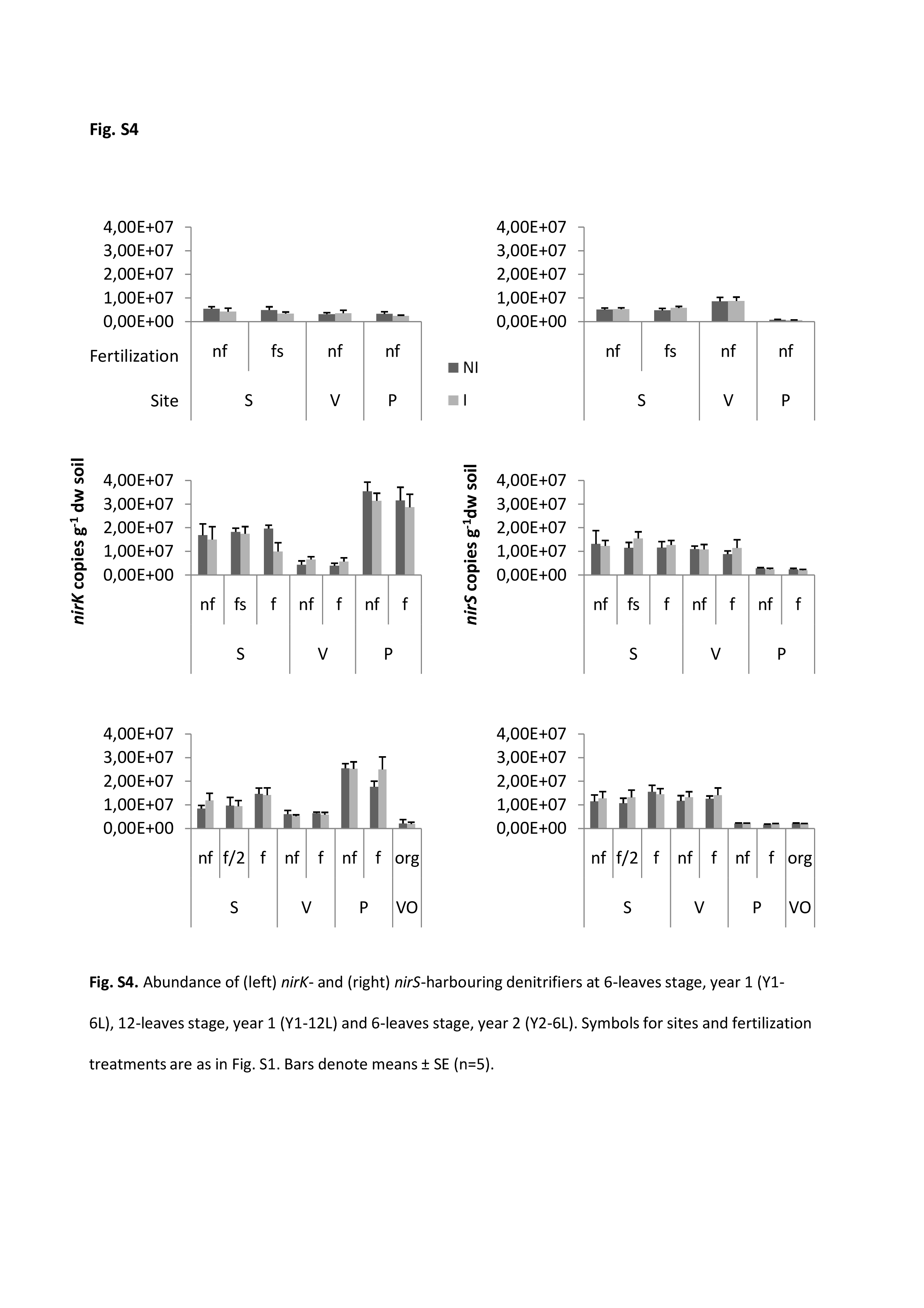

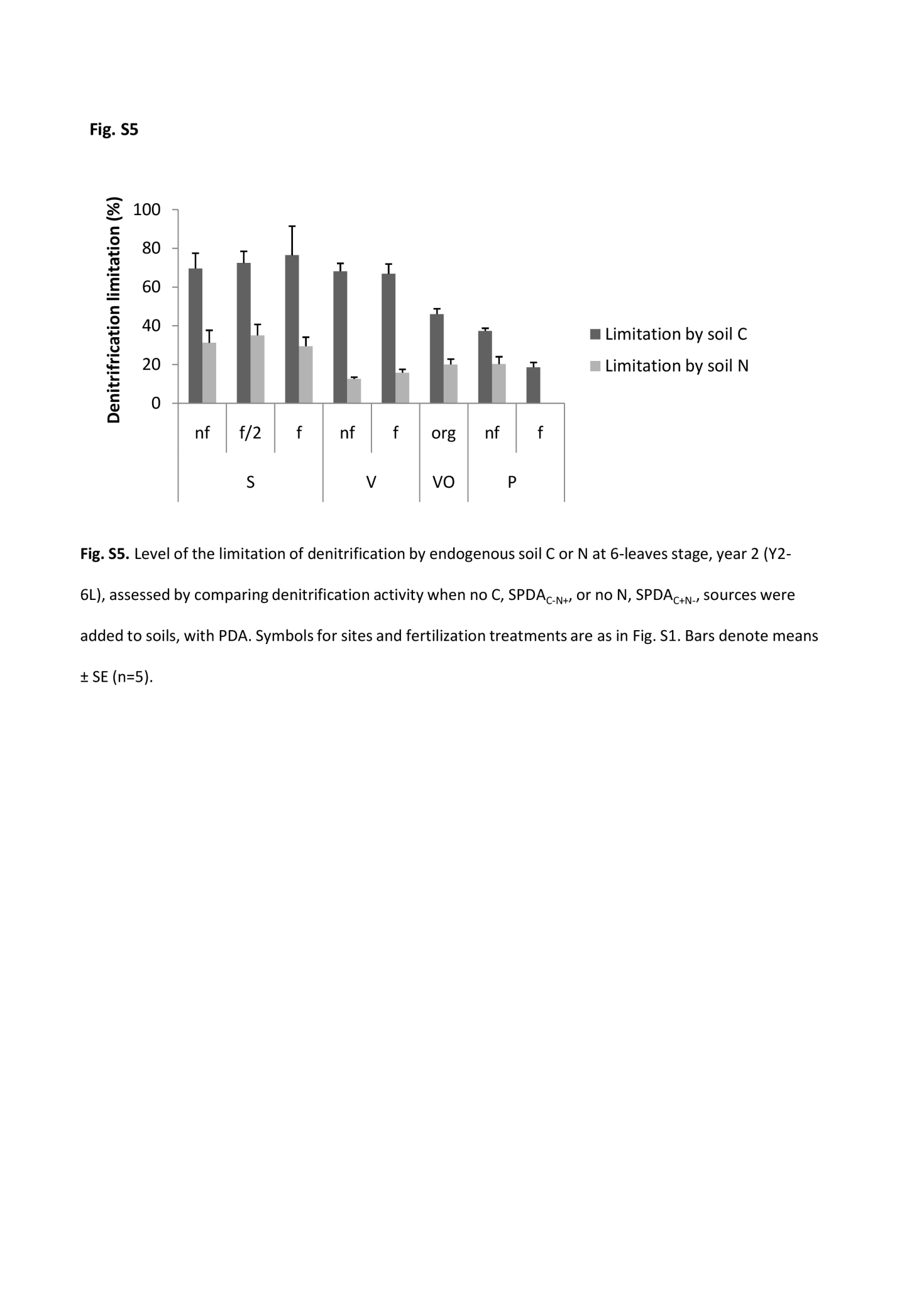

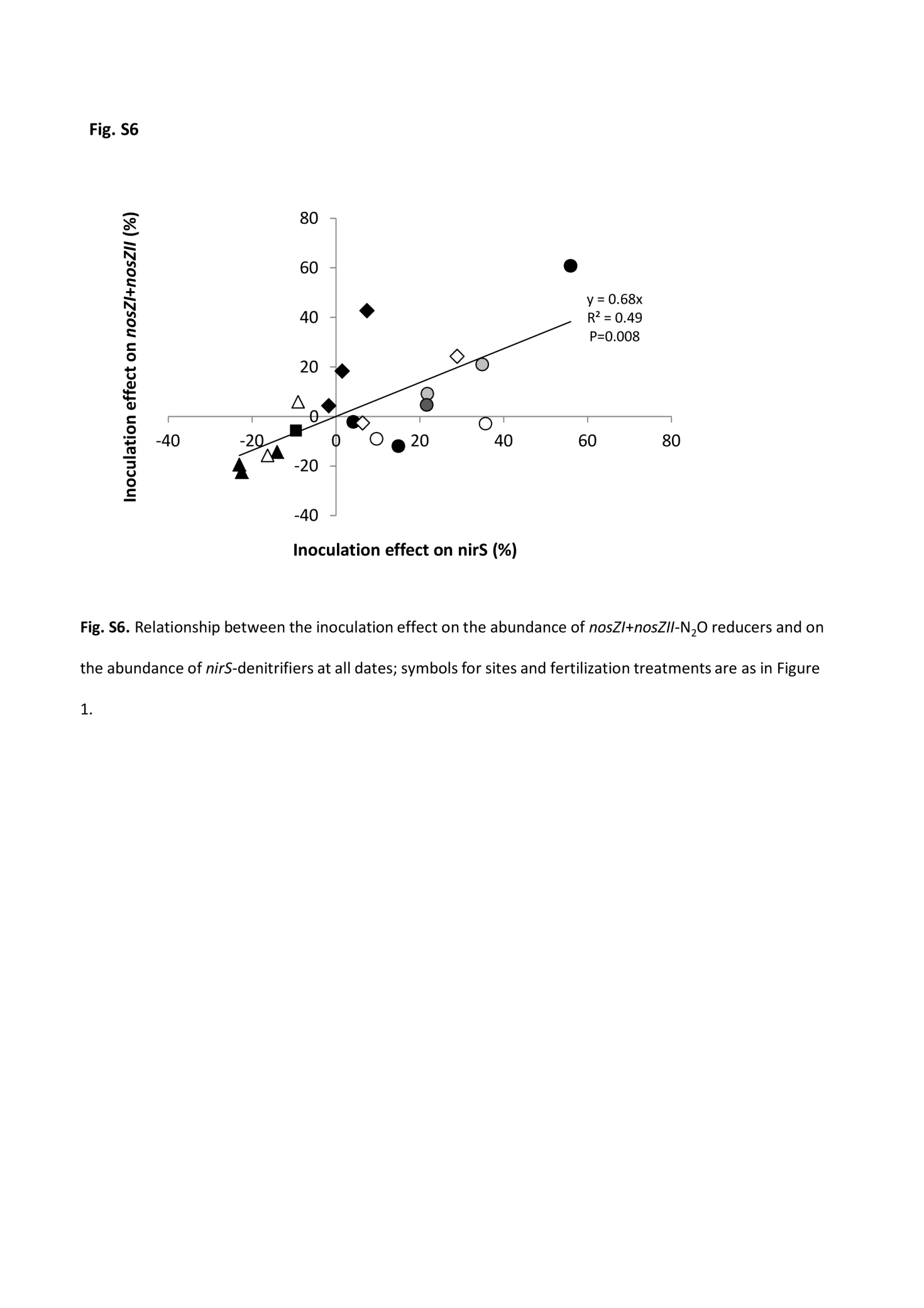

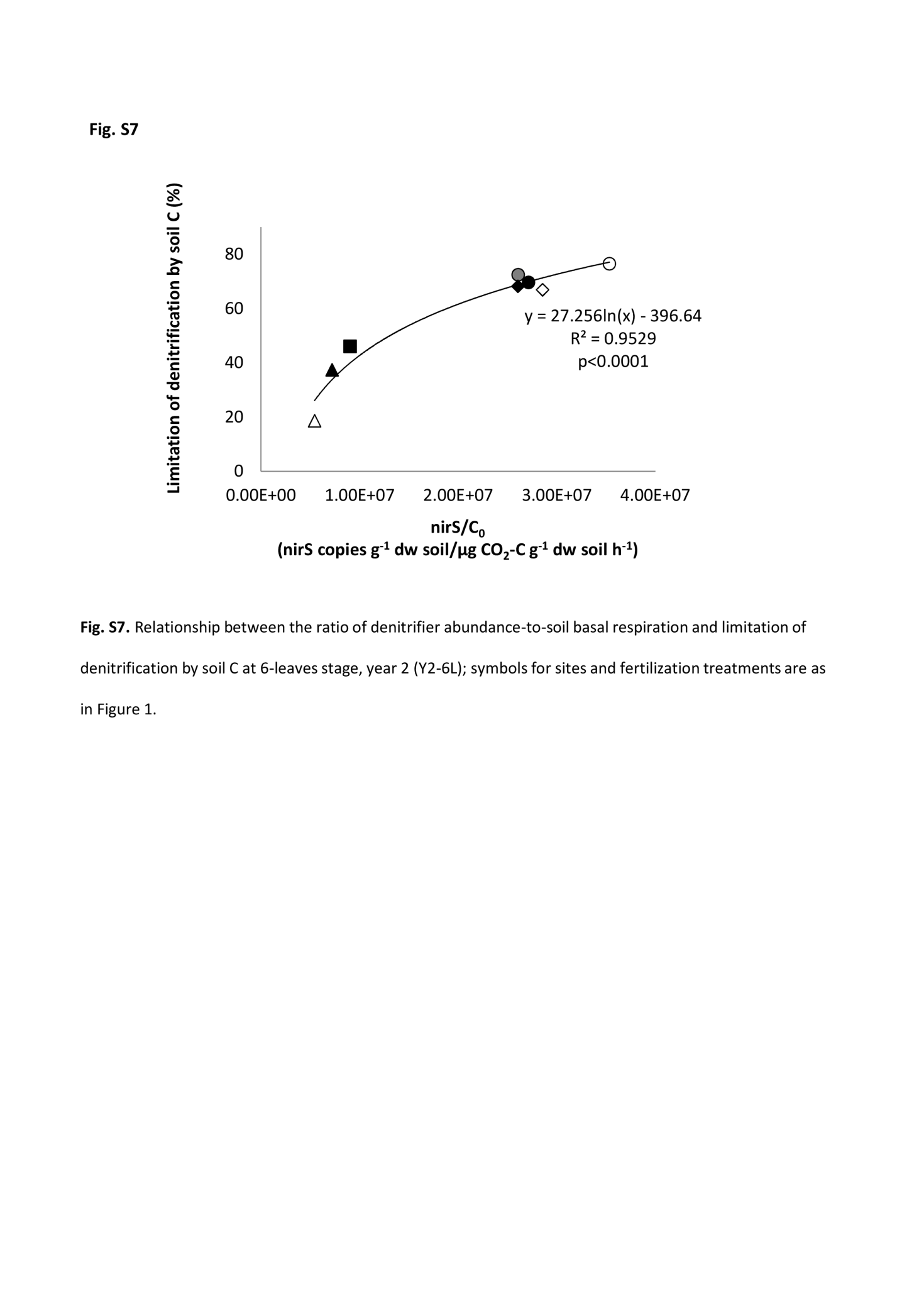

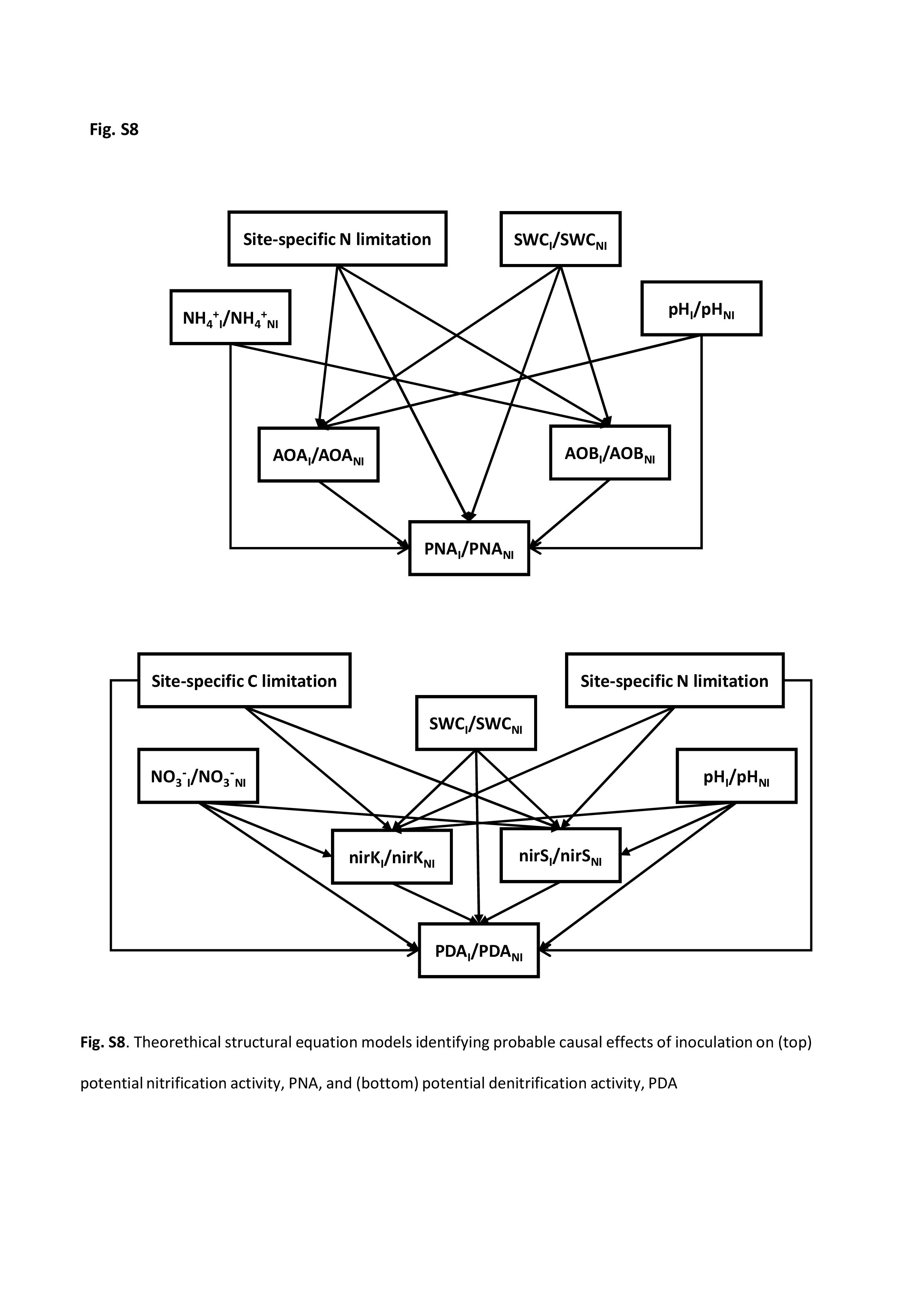

Supplement: Supplementary file 1 — Supplementary Material [file 41598_2017_8589_MOESM1_ESM.doc]
